# Aging Effects on Optic Nerve Neurodegeneration

**DOI:** 10.3390/ijms24032573

**Published:** 2023-01-29

**Authors:** Janet Coleman-Belin, Alon Harris, Bo Chen, Jing Zhou, Thomas Ciulla, Alice Verticchio, Gal Antman, Michael Chang, Brent Siesky

**Affiliations:** 1 Department of Ophthalmology, Icahn School of Medicine at Mount Sinai, New York, NY 10029, USA; 2 Vitreoretinal Medicine and Surgery, Midwest Eye Institute, Indianapolis, IN 46290, USA; 3 Department of Ophthalmology, Rabin Medical Center, Petah Tikva 4941492, Israel

**Keywords:** optic nerve, senescence, active aging, oxidative stress, glaucoma, diabetic retinopathy, neurodegeneration, neuroregeneration, inflammation, embryology

## Abstract

Common risk factors for many ocular pathologies involve non-pathologic, age-related damage to the optic nerve. Understanding the mechanisms of age-related changes can facilitate targeted treatments for ocular pathologies that arise at any point in life. In this review, we examine these age-related, neurodegenerative changes in the optic nerve, contextualize these changes from the anatomic to the molecular level, and appreciate their relationship with ocular pathophysiology. From simple structural and mechanical changes at the optic nerve head (ONH), to epigenetic and biochemical alterations of tissue and the environment, multiple age-dependent mechanisms drive extracellular matrix (ECM) remodeling, retinal ganglion cell (RGC) loss, and lowered regenerative ability of respective axons. In conjunction, aging decreases the ability of myelin to preserve maximal conductivity, even with “successfully” regenerated axons. Glial cells, however, regeneratively overcompensate and result in a microenvironment that promotes RGC axonal death. Better elucidating optic nerve neurodegeneration remains of interest, specifically investigating human ECM, RGCs, axons, oligodendrocytes, and astrocytes; clarifying the exact processes of aged ocular connective tissue alterations and their ultrastructural impacts; and developing novel technologies and pharmacotherapies that target known genetic, biochemical, matrisome, and neuroinflammatory markers. Management models should account for age-related changes when addressing glaucoma, diabetic retinopathy, and other blinding diseases.

## 1. Introduction

The optic nerve, also known as cranial nerve II (CNII), is composed of purely sensory special somatic afferent (SSA) fibers [1] and is subject to age-dependent neurodegeneration. Optic nerve injury and resultant degeneration is the root cause of permanent visual loss in multiple age-related ocular diseases, including glaucoma [2,3] and diabetic retinopathy [4,5,6], which together account for 13.5% of all blindness worldwide in adults age 50 and older [7,8] and which were estimated in 2020 to affect 179,120,00 people globally [9,10] (~the combined populations of eight Central American countries, including Mexico [11]). Age-dependent changes in the optic nerve are mediated by structural and biochemical cascades that ultimately result in neurodegeneration, cell death, and resulting permanent visual loss. Understanding the mechanisms and impact of aging on optic nerve tissues is pivotal to developing neuroprotective measures to slow and prevent optic nerve disease.

**Figure 1:**
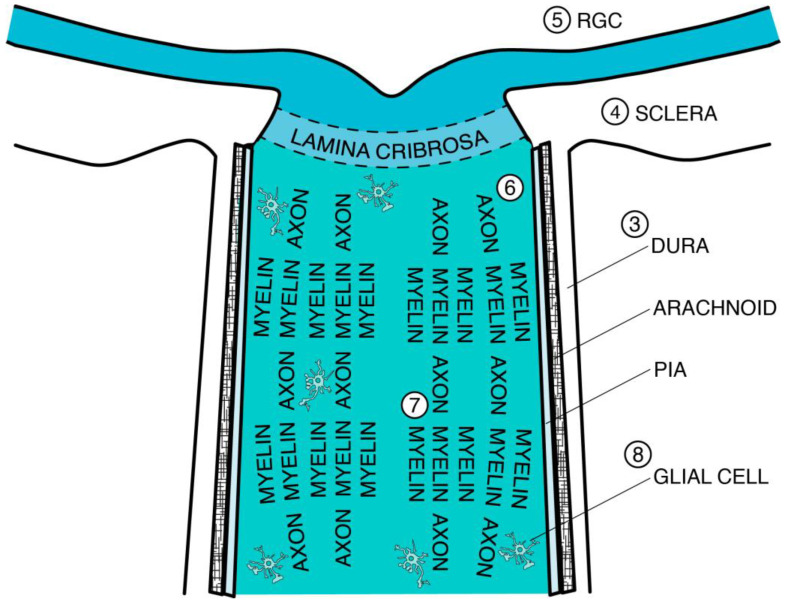
Schematic anatomical image of the optic nerve pertaining to age-dependent neurodegeneration. Numbers correspond with section headings of the present review, specifically “3. Connective Tissue,” which includes “3.4. Dura Mater,” “3.5. Arachnoid and Subarachnoid Space,” “3.6. Pia Mater and Septa,” and “3.7. Lamina Cribrosa (LC)”; “4. Decreased Optic Canal Expansion”; “5. Retinal Ganglion Cell (RGC)”; “6. Axon”; “7. Myelin”; and “8. Glial Cell.”

This review will discuss age-dependent changes in the optic nerve and resulting neurodegeneration. Specifically, aging is associated with structural changes in the optic nerve, including connective tissue thickening, stiffening, and weakening; these affect the dura mater, arachnoid (and subarachnoid space), pia mater, septa, and lamina cribrosa (LC). Resulting mechanical changes lead to decreased optic (scleral) canal expansion at the optic nerve head (ONH) and retinal ganglion cell (RGC) loss, concurrent with age-associated extracellular matrix (ECM) remodeling and biochemical RGC death. Additionally, aging is associated with axonal loss of density, swelling, lowered regenerative capacity, and metabolic dysfunction; even when axons regenerate, however, age-related failures in remyelination prevent meaningful axonal function. In response to injury, aged glial cells (astrocytes and microglia) can maladaptively react to create a neuroinflammatory microenvironment that can result in RGC axonal death and visual loss. Anatomic landmarks regarding optic nerve aging are schematically illustrated in Figure 1.

These age-associated features (summarized in Table 1) have the potential to contribute to the pathophysiology of neurodegenerative ocular disorders, particularly diabetic retinopathy and glaucoma. Further clarifying the structural alterations, biochemical modifications, and molecular changes affecting the optic nerve throughout the human lifespan can support innovative interventions against aging and associated mechanisms of optic nerve neurodegeneration. Future investigations searching for novel therapeutic targets, pharmacotherapies, and technologies hold promise for the clinical management of glaucoma, diabetic retinopathy, and other blinding diseases.

## 2. Optic Nerve Development

### 2.1. Embryology

The human optic nerve originates from neuroectoderm. The earliest signs of eye development occur at week three of gestation with the formation of optic grooves from the developing forebrain. The optic grooves then evaginate forming the optic vesicles, which eventually become the optic stalk and later on, the optic nerve. At week four of gestation, the optic vesicle invaginates to become the optic cup (and later, the retina). Grooves called retinal fissures form around the hyaloid artery and vein (later the central retinal artery and vein); these vessels become encircled within the optic nerve when the optic cup invaginates and fuses, additionally leading to the retinal outer layer (later, retinal pigment epithelium (RPE)) and the inner layer (neural retina). The neural retina contains photoreceptors, several classes of interneurons, and RGCs. RGC axons project into the optic stalk lumen, obliterating the lumen and forming the optic nerve [179].

Importantly, normal optic nerve development features an overproduction of axons during the first 15 weeks of gestation, after which approximately 50–70% of axons are eliminated between weeks 16 and 30 of gestation [60,118,119], likely mediated by apoptotic caspases (particularly caspase-3) [60]. Collagen cross-linking during development and maturation is precise, controlled, and enzymatically regulated by lysyl oxidase (LOX), lysyl oxidase-like (LOXL) enzymes 1–4, transglutaminase 2 (TG2), and peroxidasin (PXDN) [24]. 

### 2.2. Newborn to Elderly

Histologic studies of human optic nerve midpoints reveal that at birth, the optic nerve diameter is small, with an average area of approximately 2.8 sq mm– 4.27 sq mm [12,180]. The area doubles in size between birth and ages 3–4 years with a slower increase until the optic nerve reaches adulthood at age 12–15 years [180], after which the diameter remains mostly constant with an average area of 6.75 sq mm– 9.93 sq mm [12,180] for the remainder of life (ages 7–96 years). Retrobulbar optic nerve diameters are consistently an eighth larger than midpoint diameters [12]. 

## 3. Connective Tissue: Thickening, Stiffening, and Weakening

The human optic nerve’s meningeal-derived extracellular matrix (ECM) is composed of an extensive network of macromolecules, including elastin, collagen, laminin, fibronectin, hyaluronan-binding proteins, and tenascin [181,182]. Age-dependent features, which occasionally overlap with ophthalmic pathologies, alter the ECM’s delicate microarchitecture and affect the five major connective tissue elements of the optic nerve: dura mater, arachnoid (and subarachnoid space), pia mater, septa, and LC. Elastin and collagen connective tissue fibers provide mechanical strength, with biomechanical properties controlled by ECM composition, concentration, and post-translational modifications (including glycosylation, transglutamination, and crosslinking) [13]. While less prominent in the literature on optic nerve aging, alterations in other ECM glycoproteins (including the degradation of laminin) may contribute to optic nerve neurodegeneration [181].

### 3.1. Elastic Fibers

Elastic fibers are present in all connective tissue elements, primarily the lamina cribrosa (LC) followed by the dura mater. In youth, there is relatively minimal elastic tissue. At senescence, notably in ages >60 years, elastic fibers are reported to increase [12,14]. These are especially prominent in pia mater and septa of the retrobulbar optic nerve [12]. LOX oxidizes peptidyl lysine in collagen and elastin to become peptidyl aldehydes that spontaneously condensate, post-translationally modifying them to form covalent and insoluble cross-linkages [15].

Notably, while vascular, dermal, and pulmonary tissues experience age-dependent decreases in elastic fibers [16], age-dependent changes in the quantity of elastin at the optic ONH and LC are uncertain, with some data suggesting increased elastic fibers with age in these regions [12,14]. What is ubiquitous in connective tissue aging is a decrease in the quality of elastin and an increase in collagen strength [16,17,18,19], resulting in mechanically weaker fibers [20].

Investigations in arterial tissue biomechanics indicate that ECM organization is more indicative of an artery’s mechanical properties than composition [16,21,22]; one such example is that the collagen/elastin ratio is a poor predictor of arterial wall mechanics [16]. While current reviews of ONH biomechanics imply that ECM composition and concentration are critical to the ONH mechanical properties [13,23], there is an opportunity for further investigation to determine the relative importance of ECM composition and organization at the ONH. 

### 3.2. Collagen Fibers

While collagen cross-linking during development is precise, controlled, and enzymatically regulated [24], age-dependent ECM collagen changes participating in optic nerve degeneration may be driven by elevated LOX expression (overactive enzymatic activity) or advanced glycation end products (AGEs) and their receptor for AGE (RAGE) (unregulated, non-enzymatic) [23,25,26,27]. Abnormal collagen crosslinking induced by ECM remodeling and modified tissue biomechanics can promote age-dependent neurodegeneration, including glaucoma [23]. 

#### 3.2.1. Elevated Lysyl Oxidase (LOX) Expression

LOX is the primary enzyme regulating collagen cross-linking during development and maturation; elevated LOX expression, however, is associated with tissue fibrosis [15,26,27]. LOX oxidizes peptidyl lysine in collagen and elastin to become reactive peptidyl aldehydes that spontaneously condensate, post-translationally modifying them to form covalent and insoluble cross-linkages [15]. Additionally, because LOX is located intracellularly and intranuclearly, its multifunctional activities have been implicated in cellular development, tumor suppression, motility, senescence, and may even have a role in cell adhesion and growth control [27]. 

#### 3.2.2. Advanced Glycation End Products (AGEs) 

AGE accumulations and their multi-ligand RAGE expression are well-documented in aging tissues and are the major cause of cellular dysfunction in elderly collagenous tissue [26]. Notably, AGEs have been implicated in various age-dependent pathologies including atherosclerosis, Alzheimer’s disease, infection [25,183,184], presbycusis [185], diabetic retinopathy [101], glaucoma [25], and even age-related macular degeneration [186]. 

AGEs irreversibly form when proteins have extended exposure to reducing sugars, causing non-enzymatic glycation of amino groups by the Maillard reaction [25,184]. Early-stage glycation results in Amadori compounds, whereas late-stage results in AGEs. AGEs preferentially form on long-lived macromolecules (including collagen, proteins, lipids, and nucleic acids), were primarily detected in retinal and ONH ECM, and exhibit pigmentation and fluorescence [25]. While collagen normally crosslinks at specific sites on molecule ends, AGEs crosslink throughout the length of the molecule [187] after several chemical effects, including oxidation [25]. 

AGE’s receptors are located on numerous cells that normally function to protect and repair, including monocytes, macrophages, endothelial cells, pericytes, microglia, and astrocytes [25]. One of the most notable receptors for AGE is RAGE. AGE binding to receptors, including RAGE, leads to the release of transforming growth factor beta (TGF-β), tumor necrosis factor-alpha (TNF-α), interleukin-6 (IL-6), and other profibrotic and proinflammatory cytokines [25]. Molecularly, AGEs may inhibit normal cellular functions, such as axonal transport and intracellular protein traffic [188]; globally, AGEs may induce rigidity, particularly at the LC [25].

#### 3.2.3. Receptor for AGE (RAGE)

There is a proposed “two-hit hypothesis” for the interactions with RAGE, whose ligands include AGE; amyloid fibrils, including amyloid-β peptide (Aβ); amphoterins; and S100/calgranulins [184]. Ligands first bind RAGE and initiate cellular activation via slow endocytosis and lysosomal degradation [189]. The next “hit” features the RAGE-mediated activation of effector cells that interferes with normal protective and reparative processes and may cause superimposed lipoprotein accumulation, bacterial invasion, and ischemic stress [184]. RAGE reliably induces cell dysfunction by generating reactive oxygen species (ROS) [184] and activating p21^ras^ and phosphatidylinositol 3-kinase pathways, mitogen-activated protein kinases [190,191], Rho family small GTPases (especially Cdc42 and Rac, implicated in RAGE-mediated changes in cell migration) [102].

Together, these two “hits” create a pro-inflammatory environment favoring tissue destruction. Unlike receptors that downregulate expression with increased ligands, such as the low density lipoprotein receptor (LDLR) [192], RAGE increases expression with increased ligands and propagates cellular dysfunction [184], especially in neurons with cyclic-AMP response element binding protein (CREB) activation and elevated chromogranin expression [102,193]. AGE accumulation may, therefore, modify ECM physicochemical properties, where ECM can become less soluble, less susceptible to enzymes, less thermodynamically stable, less mechanically strong, and less flexible [25,103,194,195,196,197].

#### 3.2.4. Other Molecular Mechanisms

Other molecular mechanisms of ECM remodeling associated with fibrosis have also been postulated to be influenced by calcium and lipid accumulation, along with proteolysis by matrix metalloproteases (MMPs) [22,23]. Calcium and lipid accumulation are less well-studied than elevated LOX expression and AGE/RAGE expression and provide an opportunity for further investigation. MMPs will be discussed shortly.

#### 3.2.5. Mechanical Considerations

While there are several mechanical considerations affecting collagen, including microstructural crimp and mechanical weakening [38,198], these have been primarily studied in the LC and sclera and will be discussed in their respective sections (“3.7. Lamina Cribrosa (LC)” and “4. Decreased Optic Canal Expansion”). 

### 3.3. Laminin

Laminin, an adhesive glycoprotein known for connecting intracellular and extracellular compartments, is primarily found on basement membranes [199]. Age-dependent degradation of laminin may contribute to optic nerve neurodegeneration and related disorders [181,182] along with age-related vitreoretinal disorders, such as posterior vitreous detachment [200].

#### Matrix Metalloproteases (MMPs)

In the optic nerve, proteolytic MMPs are associated with collagen fibrosis [22,23] and gelatinolytic MMPs with laminin degradation and RGC death [201]. Specifically, matrix metalloproteinase gelatinase B (MMP-9, *GelB*) has been implicated in retinal degeneration [201,202]. In mice, excitotoxic kainic acid intravitreal injection caused dose-dependent MMP-9 upregulation, astrocyte activation, decreased laminin immunoreactivity, and resulting RGC death (attenuated by injecting non-NMDA receptor antagonists CNQX and NBQX) [202]. Following optic nerve ligation, *GelB*-deficient (*GelB^−/−^*) mice did not exhibit significantly reduced RGCs in the ganglion cell layer nor produce terminal deoxynucleotidyl transferase biotin-dUTP nick end labeling (TUNEL) positive cells in injured retinas [201]. The protective nature of MMP-9 deficiency, given the lack of apoptotic markers following optic nerve injury in MMP-9-deficient mice [201], is promising for the development of matrisome technologies and pharmacotherapies to prevent RGC death following injury [182]. Further investigations on MMPs in human optic nerves are needed to confirm results.

### 3.4. Dura Mater

There are two layers of the human optic nerve dura mater: inner and outer. The inner layer of dura has more collagen and elastin than the outer layer [17]. Recall the dura has many elastic fibers, second only to the LC, and given the histologic study of optic nerve connective tissue (in contrast to other tissues [16]), elastic fibers may increase at senescence [12,14]. 

The thickness of dura averages 0.3 mm and increases at the ONH merging with peripapillary sclera (PPS) [28]. Dura mater doubles in thickness after birth through age 1 (average 0.188 mm), and doubles again from age 1 to optic nerve adulthood at ages 12–15 (average 0.375 mm) [12]. The circumference enlarges, leading to the redundancy of the membrane and subsequence wrinkling, most prominent at age 60–70 near the globe of the eye. Dural calcifications are possible and can reach up to 4 mm in diameter [12]. 

Intriguingly, brain dura becomes less stiff with aging. Elastic modulus, tensile strength, and the maximum strain all decrease with age, irrespective of sex and the side of the cranium examined [29]. 

### 3.5. Arachnoid and Subarachnoid Space

At youth, the arachnoid membrane is described histologically as thin and delicate, and subarachnoid space features minimal slender trabeculae [12]. Middle age, accelerating during age 60–70 years, features numerous coarse (usually rounded, then hyalinized, and occasionally calcified) trabeculae. The arachnoid outer membrane thickens with more prominent arachnoid granulations that may have a fibrous core [12]. 

Cerebrospinal fluid pressure (CSFP) decreases with age [30], which has been linked to normal tension glaucoma with the mechanism still unclear. This could be due to a higher-than-normal pressure gradient across the ONH, between intraocular pressure (IOP) and CSFP [31]. The increased pressure difference could alter the shape of the LC and, thus, mechanically damage RGC axons [31]; however, recent evidence suggests CSFP changes may modulate strain at ONH independently [32]. The link between CSFP and age-dependent normal tension glaucoma could also be due to a rise in toxic protein concentrations [33]. 

### 3.6. Pia Mater and Septa

Pia mater at birth is thin, doubles in thickness after birth through age 1 (average 0.0469 mm), remains consistent through age 55, and gradually becomes thicker at senescence (average 0.938 mm after age 60) as pia expands into a solid fibrous sleeve encircling the optic nerve [12]. 

As septa are extensions of pia mater into the optic nerve axons, they experience identical changes to pia. In septa, age-dependent changes are most substantial in the retrobulbar nerve [12].

### 3.7. Lamina Cribrosa (LC)

As the connective tissue is the richest in elastic fibers [12], the mechanosensitive LC may be the most well-studied connective tissue of the optic nerve [34,35,36].

The LC ECM stiffens with age, reportedly due to age-dependent features including significant collagen deposition [18,19], increased cribriform plate rigidity [35], unique vascularity [37], and thicker astrocyte basement membranes [18,19]. Each warrants its own section, given the literature investigating these four age-dependent features is extensive.

#### 3.7.1. Collagen Deposition

The LC is composed of constituent proteins (including elastin and collagen types I, III, IV) and longitudinal fibers in a proteoglycan matrix [203]. As with ECM in many aging tissues, the quality of elastin decreases while collagen deposition increases [16], and collagen is fundamental in mediating increased LC stiffness in vivo [34]. As discussed above (see “3.2. Collagen Fibers”), age-dependent collagen deposition may be mediated by AGE and RAGE. On the molecular level, AGE and its receptors may inhibit normal cellular functions and promote cellular dysfunction and destruction [184,188]; globally, AGEs may induce LC rigidity [25].

Stress-related modified ECM composition and organization are mediated by mechanical cues and subsequent release of signaling molecules, with the ultimate goal of limiting further strain [34,204]. Namely, myofibroblast contraction both activates and releases latent pro-fibrotic TGF-β from the ECM LC cells [204]. An intracellular cascade follows mechanotransduction [34]. These changes are particularly notable in aged LCs, where increased tissue stiffness and increased LC stiffness have increased prevalence in elderly individuals [34]. While the ensuing overall LC stiffening stabilizes the LC structure by reducing strain [34,35], the structural changes and increased underlying ECM stiffness may impede the already vulnerable singular neurovascular transit at the LC [37,205], causing RGC death and subsequent vision loss [34]. 

Intriguingly, in vitro optic nerve cells from the LC express biochemical similarities to myofibroblast cells, namely constitutive α–smooth muscle actin expression, elastin, collagen type I, and fibronectin [206]. In vitro LC cells also upregulate TGF-β1 (induces differentiation of fibroblasts into myofibroblasts [207,208]) and MMP-2 (degrades elastin and collagen [23]) in response to cyclical strain [209,210].

#### 3.7.2. Increased Cribriform Plate Rigidity

The age-dependent stiffening leading to decreased mechanical compliance and resilience of the LC implies that stress applied to the aging LC may result in increased LC plastic flow [35]. Known as the irreversible deformation after the reversible elastic limit is reached, plastic flow permanently alters the tissue such that removal of stress does not allow the strain to return to zero; furthermore, plastic flow increases until the tissue’s failure strength [35]. Age-dependent rigidity of the cribriform plate then continually reintroduces stress and, therefore, the strain on the LC, increasing plastic flow towards LC tissue strength failure. In contrast, pliable and misaligned cribriform plates in newborns do not increase plastic flow [35]. 

Notably, it has been posited that a genetic or environmental susceptibility to ONH stiffening at younger ages may be a mechanism of predisposition to glaucoma [34]; however, the relationship has not been proven and remains unclear [35,61,211].

#### 3.7.3. Unique Vascularity

The LC is the only location in the central nervous system (CNS) where capillaries do not directly feed astrocyte processes [205]. Instead, RGC axons are believed to receive singular nutrition from local astrocytes [37], where collagen and vascular networks are visibly distinct [212]. As a result, there are many possible vulnerabilities in the neurovascular nutrient diffusion to the LC and RGC axons [37]. 

One major barrier to nutritional access is ECM remodeling in the form of age-dependent changes to LC beams [37]. LC thin beams and thick beams have differing collagen microstructure and resulting properties; notably, thin beams have increased crimp amplitude, whereas thick beams have increased crimp waviness and tortuosity. As a result, thin beams may stiffen at lower IOP than thick beams to support comparable stress of IOP-induced force [213]. Newly published research from Waxman et al. in 2022 indicates that collagen and vasculature in the LC are separate, where 78% of LC beams did not contain vessels, and 79% of vessels were located inside beams [212]. These findings refute the previously held belief that each LC beam has a vessel within the beam [212,214]. Furthermore, vessels external to beams may be more mechanically vulnerable to compression by elevated IOP than vessels within beams [212]. 

#### 3.7.4. Thicker Astrocyte Basement Membranes

Tensile strain affecting LC beam stiffening may additionally cause the astrocyte basement membrane to thicken, in addition to endothelial cells of the vasculature, which could interfere with the singular nutrition from local astrocytes [37,205]. Because the LC’s ability to adapt to changes in stress and strain may also be mediated by integrins [108], it is conceivable that integrins also impact astrocyte basement membrane thickening.

Integrins α2β1, α3β1, α6β1, and α6β4 may be key attachment molecules (via laminin) for astrocytes to collagen type IV basement membranes. These integrin-based focal mechanoreceptors, which exist in the ONH of primates, may allow for ECM modification following stress within or anterior to the LC. Notably, these modifications may also be implicated in age-dependent glaucomatous neurodegeneration [108]. Additionally, integrins have an active role in CNS vascular pathology [104,147,148,215,216], including ocular neovascular disease.

## 4. Decreased Optic Canal Expansion

Decreased optic (also known as scleral) canal expansion is mediated by the effects of the LC and sclera on the ONH. As molecular mechanisms for LC stiffening are previously discussed, this section centers on biochemical and mechanical changes at the sclera and ONH impacts.

### 4.1. Scleral Stiffening

As investigation findings differ based on methods, age, and species, existing literature debates the exact structural and compositional changes of the aging sclera [39]. There appears to be a consensus that the sclera likely stiffens and may be due to accumulations of non-enzymatic collagen crosslinks (also known as AGEs) and increased quantity of tropocollagen molecules per fibril [39]. However, some studies note scleral thinning with age [40,41,42] (occasionally concurrent with stiffer response [42]) while others note no age-related histological differences in scleral thickness [43]. Compared to females, males exhibit a thicker anterior sclera [44,45,46,47,48]; statistically significant associations between thickness and biological sex remain to be confirmed at the posterior sclera [42,49]. There is an opportunity for a final consensus, including explanations for these varied findings, in future research.

The sclera is the eye’s primary load-bearing connective tissue and features dynamic ECM remodeling and biochemical responses [39]. Specific age-dependent protein changes in the human sclera include decreased elastic fibers after age 20 [50]; decreased decorin and biglycan levels after age 40 [51]; increased cross-sectional collagen fibril area [52] (which may be due to AGE accumulates [53]); increased mean collagen fibril radius; and increased intermolecular lateral spacing [54]. Additionally, wide-angle X-ray scattering (WAXS) measurements of aged human sclera revealed substantial collagen fiber alignment with no changes in orientation [55], yet small-angle light scattering (SALS) measurements did not find significant age-related variations in collagen fibers [49,56]. Though the local orientation of collagen fibrils changes throughout the sclera, there were no significant variations with age [49]. 

Scleral stiffening may be due to an age-dependent decline in collagen crimp parameters [31], namely waviness, tortuosity, and amplitude [38]. Crimp limbus morphology had the most dramatic age-related changes, where newborns had the highest crimp (which decreased fastest with age) and elderly individuals had the lowest crimp [38]. This is consistent with experimental confirmation that microstructural crimp is the source of the sclera’s nonlinear biomechanical behavior [57]. Additionally, scleral stiffening could be induced by glycosaminoglycan modifications [58,59].

Finally, the scleral shape at the boundary with the ONH has age-dependent changes. Elderly individuals have a more pronounced v-shaped peripapillary sclera (PPS), where the PPS angle increases an average of 0.233 degrees/year and the peak is pointed toward the orbit [217]. 

Of the elements affecting age-dependent optic nerve neurodegeneration, the structure and biomechanics of the aging sclera may be the area in most need of further investigation. 

#### Central Serous Chorioretinopathy (CSCR)

CSCR, characterized by localized serous retinal detachment, has known association with endogenous and exogenous corticosteroids and sympathomimetic compounds, along with conditions that may contribute to elevated stress (including pregnancy, poor sleep quality, shift work schedule, Type-A personality behavior, and psychiatric symptoms) [218]. Scleral thickening has been proposed as a risk factor for CSCR, perhaps due to altered scleral wall mechanics, impacts on vascular hemodynamics, and resulting abnormal choroidal venous outflow (as represented by venous overload choroidopathy) [45]. Consistent with this hypothesis are recent findings that regional thickness in the posterior and equatorial sclera are statistically significant predictors of CSCR (p=0.001) [219]. As the average age of patients with CSCR is most consistently reported to be 45-51 years old [218], CSCR may offer insight into age-dependent scleral changes. 

### 4.2. Optic Nerve Head (ONH) Impacts

The age-dependent stiffening of both LC ECM and sclera may alter the biomechanical environment of the ONH [35,61,62,63]. 

Stiff sclera is associated with increased ganglion cell loss [41,64,65]. This may be due to a stiffer sclera preventing optic canal expansion; when IOP is elevated and directly acts on LC’s anterior surface, decreased canal expansion prevents LC stretching and displaces the LC posteriorly [66]. These findings are consistent with reported ONH impacts in the 2018 Biomechanics of the Eye textbook, which states that the size of the optic canal significantly impacts retrolaminar optic nerve deformation [20]. 

Intriguingly, multiple regression analyses on the optic canal area and axon count reveal that when corrected for age, eyes with smaller optic canal area have a larger quantity of axons [67]. Optic nerve axons are known to diminish with age [12,60] (see section “6. Axon”). Further research is indicated to investigate the mechanism, such as whether a smaller scleral canal area is associated with a less stiff sclera, larger expansion, and possibly less ganglion cell loss. 

## 5. Retinal Ganglion Cell (RGC): Loss and Injury

While stiff sclera and decreased optic canal expansion are associated with mechanical RGC loss [41,64,65], RGCs also experience age-dependent biochemical loss. These are largely mediated by caspase-dependent apoptosis [60], histone lysine methylation [68], and HAT and HDAC deacetylation [73]. Importantly, the visual loss seen clinically in diabetic retinopathy and glaucoma (two age-dependent ocular neurodegenerative pathologies) is due to RGC destruction and will be discussed here. 

### 5.1. Embryonic

Developmental loss of RGCs is primarily induced by caspase-3 neuronal pruning [60]. Normal development overproduces RGCs, necessitating the elimination of approximately 50% of RGCs shortly after cell birth. Active caspase-3 selectively localizes to TUNEL-positive RGCs in chick embryos [69]; inhibiting caspase-3 increased the quality of RGC by approximately 50% and additionally increased axons and ganglion cell layer (GCL) thickness. Developmental RGC reduction can also be experimentally prevented by broad-spectrum caspase inhibitors such as boc-D-fmk [69,70]. BarH-like homeobox 2 (BARHL2, *Barhl2*), part of the Bar-class HD (BarH) gene family, regulates caspase-3 activation and is essential to preserve normal RGC complement during mouse development [71]. 

Histone lysine methylation (e.g., H3K9me2 and H3K27me3) and regulation by respective histone methyl transferases G9a (*EHMT2*) and enhancer of zeste homolog 2 (Ezh2, *EZH2*) also occur during in the developing mouse retina [68]. Transcriptional gene control and microglial phagocytosis additionally limit RGC density. Complement proteins mark RGC for microglial phagocytosis; specifically, removing complement receptor 3 (CR3, a microglia-specific receptor) increases RGC numbers [72]. 

### 5.2. Age-Dependent Loss of RGC and Axons

Aging is associated with the loss of RGC and axons [12,74]. The proportion of neuronal tissue in the retina nerve fiber layer (RNFL) thickness decreases with age [74]. Age-dependent loss of RGC and axons are mediated by canonical (caspase-dependent) and non-canonical (caspase-independent) biochemical mechanisms. 

Caspases, or cysteine aspartate proteases, are divided into two major phylogenic subfamilies: IL-1β-converting enzyme (inflammatory, caspase-1, -4, -5, -11, and -12) and mammalian counterparts of cell death gene *ced-3* (apoptotic, caspase-2, -3, -7, -8, -9, and -10) caspases. The apoptotic caspases can be further separated into initiator and executioner caspases. Specifically, initiator caspases (caspase-2, -8, -9, and -10) activate executioner caspases (caspase-3, -6, and -7) through catalytic cleavage of their activation domain [60]. Interactions between TNF receptor members (Fas/CD95 receptor), Fas-associated protein with death domain (FADD), and pro-caspase-8 form death-induced signaling complex (DISC). Once caspase-8 is activated, it cleaves and thereby activates executioner caspase-3, -6, -7. Caspases can be both initiator and executioner caspases [60], including caspase-2 (crucial in optic nerve injury [75,76,77,78,79]). Additional mechanisms of caspase action are extensive; due to the length of this manuscript, the authors will leave caspase impact on optic nerve neurodegeneration at this level of discussion.

Canonical (caspase-dependent) and non-canonical (caspase-independent) cell death are involved in RGC degeneration. Other mechanisms of RGC loss are via histone acetylase (HAT) and histone deacetylase (HDAC) [73]. Increased HAT and HDAC activity lead to deacetylation and neurodegeneration. HDAC inhibitors, such as RGFP966 or conditional knockout of *Hdac3*, offer RGC protection. In addition to neuroprotection, HDAC inhibitors reprogram chromatin through modulating p53, p300/CREB binding protein (p300/CBP), and p300/CBP-associated factor (P/CAF) [73] and promoting neuroprotection [80,81]. 

### 5.3. Aging and Injury

As the optic nerve ages, there is an accelerated loss of RGCs after optic nerve injury [82]. RGC loss is likely mediated by upregulated *DLK* gene, CaMKII-CREB signaling interference, caspases, and HDAC upregulation, though additional (albeit less robustly reported) biochemical mechanisms likely contribute. 

The most effective protection of RGCs after optic nerve injury reported thus far is from *DLK* deletion [83,84] or CaMKII-CREB [85,86] enhancement, which offer long-lasting protection for the vast majority of RGCs. Deleting the dual leucine zipper kinase (*DLK*) gene, which regulates Jun N-terminal kinase (JNK)-JUN-dependent death of RGCs following injury, does not impact RGC embryonic development [84]. Enhancing Ca/calmodulin-dependent protein kinase II (CaMKII) or its downstream CREB has been found to promote RGC survival, both following injury and in the uninjured retina [86]. 

Caspases feature inflammatory and apoptotic phylogenic subfamilies and act via apoptosis (non-inflammatory regulated cell death) and pyroptosis (highly inflammatory regulated cell death, also called caspase-1-dependent cell death) [60]. Notably, cleaved apoptotic caspase-2, -8, -9, -3, -6, -7, and inflammatory caspase-11 and -1 have been detected in RGC after crush or axotomy [60]. Caspase-2, in particular, has a central role in mediating the effects of injury, as it is exclusively located in RGC after optic nerve injury [75,76,77,78,79]. Inhibiting caspase-2 using short interfering RNA (siRNA) provides excellent neuroprotection after axotomy [75,76,77]. 

Additionally, HDAC are upregulated after acute optic nerve injury, causing increased HDAC expression, increased deacetylation, and increased RGC loss [73]. Conditional knockout of the *Hdac3* gene (encodes HDAC3) or administering RGFP966 led to dose-dependent block of HDAC3 activity and improved RGC survival by preventing nuclear atrophy and apoptosis [73,87]. (Of note, RGFP966 is an HDAC3 inhibitor with IC50 of 0.08 μM in a cell-free assay, and exhibits a greater than 200-fold selectivity over other HDAC [87].) While the conditional knockout of *Hdac* provided neuroprotection, targeted removal was not sufficiently potent to protect RGCs and axons in a mouse model [87]. HDAC3 inhibition also prevented RGC loss in aged or chronic glaucomatous mouse models, highlighting the importance of DNA acetylation as a neuroprotective [87,88]. Importantly, as HDACs are involved in many cellular processes, implementing HDAC3 inhibitor therapy to prevent RGC loss must be done with optimal precision to prevent adverse effects (including thrombocytopenia, neutropenia, anemia, fatigue, and diarrhea) [89,90].

### 5.4. Mitochondrial Dysfunction in RGC

Age-dependent changes in mitochondria include decreased activity and increased oxidative damage [91,92]; the RPE and neural retina are most vulnerable. These mechanisms of mitochondrial dysfunction are associated with worsened vision and may contribute to the pathophysiology of the aging retina [92]. Notably, mitochondrial dysfunction mediates mitochondrial dysfunction-induced senescence (MIDAS), a subtype of senescence [93].

The electron transport chain and oxidative phosphorylation functionally decline with age [92]. As the human retina has the highest oxygen-utilization rates in the human body [94], increased ROS generation damages proteins, lipids, and mitochondrial DNA (mtDNA) [92]. Along with the age-dependent worsening of mtDNA repair pathways (characterized by loss of poly (ADP-ribose) polymerase 1 (PARP1), mutY homolog (MYH), and endonuclease III homologue 1 (NTH1) in aged rodents) [91], oxidative stress increases genetic mutations [92]. 

#### Reactive Oxygen Species (ROS)

In addition to damaging mtDNA through strand breaks and base oxidation [220], ROS are known drivers of the physiologic decline and phenotype of aging [128,129]. Age-related alterations in redox homeostasis prevent ROS reduction and cause intracellular damage, oxidative stress, and an increased likelihood of age-dependent pathology [128,220,221]. Oxidative insult also increases vulnerability to neurodegeneration of the optic nerve and is associated with both primary and secondary glaucoma [220].

The transcription factor NRF2 has been implicated in the pathophysiology of aging [128]. Nuclear factor (erythroid-derived 2)-like 2 (NRF2) maintains and restores redox homeostasis: the former through the NRF2-Kelch-like ECH-associated protein 1 (KEAP1) signaling pathway, and the latter through upregulating the antioxidants glutathione (target genes *GCLC*, *GCLM*) and thioredoxin (target genes *TXN1*, *TXNRD1*) [128]. NRF2 experiences well-documented age-dependent decline, which prevents adequate ROS reduction and leads to unchecked oxidative stress [128]. Nucleic acid oxidation, if unrepaired, can lead to cellular dysfunction, oncosis, necrosis, or maladaptive apoptosis [220]. 

### 5.5. Autophagy

Autophagy, or catabolic lysosomal “recycling” of intracellular material, experiences age-related decline. Mouse retinal models exhibit decreased mRNA expression of autophagy regulators, including autophagy-related protein 7 (Atg7, *ATG7*) and Beclin-1 (*BECN1*) [95]. In the same study, aged mice modeled with mild autophagy deficiency were more vulnerable to RGC loss and axonal insult in vivo following optic nerve injury than young mice, perhaps due to oxidative stress [95]. Autophagy is associated with normal tension glaucoma via *Optn*, *Tbk1*, and *Opa-1* genes [95].

Notably, there is increasing evidence that increased autophagy is neuroprotective [95,96]. FDA-approved autophagy activators (especially the AMPK inducer and mTORC1 inhibitors) decrease ocular disease severity in preclinical and clinical trials [96]. 

### 5.6. Melanopsin-Expressing RGC (ipRGC)

Through expressing the photopigment melanopsin, intrinsically photosensitive RGC (ipRGC) autonomously phototransduce; even double knockout mice lacking functional rods and cones (*Gnat1^−/−^ Cnga3^−/−^*) are capable of brightness discrimination [97,98]. These ipRGC significantly contribute to pupillary light reflex and circadian photoentrainment, both of which exhibit age-dependent changes [99,100]. The effects of aging on optic nerve neurodegeneration (including RGC loss [74]) and ipRGC (including decreased density, decreased plexus, and lipofuscin accumulation) may contribute to age-related circadian dysfunction [100]. Additionally, melanopsin-expressing RGC loss at the ganglion cell layer (GCL) and inner nuclear layer (INL) may vary with ocular pathology; examples include ipRGC loss in patients with glaucoma (notable loss in GCL with INL sparing) and diabetic retinopathy (loss in both GCL and INL) [100].

### 5.7. Diabetic Retinopathy (DR)

DR is an age-related disease with vascular and neurovascular components [6]. Vascularly, the retina has two blood supply sources (choroid and central retina artery plexus), where the central retina artery plexus supplies half of the inner retina [105]. RGC (located in the inner retina) are more sensitive to the hypoxic challenge that occurs during diabetes mellitus [106].

In addition to a decreased oxygen supply, numerous abnormal metabolic pathways are triggered by hyperglycemia; these include pathologic polyol and hexosamine pathways, de novo diacylglycerol-protein kinase C (DAG-PKC) synthesis, and free radicals [107]. All lead to the neuron apoptosis detected in RGCs in patients with DR [4,61]. Notably, accumulation of AGEs [107] and upregulation of RAGE are prominent in retinal Müller glial cells in diabetic retinopathy [101,102,103], leading to pro-inflammatory responses. Blocking RAGE may be clinically relevant in slowing the progression of diabetic retinopathy [101].

As RGC degenerate early in the disease, patients with DR have reduced RNFL thickness compared to healthy controls [5]. Diabetic mice feature age-dependent RGC loss. Furthermore, TUNEL-positive RGC are increased in rats and humans with diabetes, along with cleaved caspase-3, caspase-9, Fas, and bcl-2-like protein 4 (Bax) localized to RGC [60]. Integrins alpha v beta 3 and alpha v beta 5 may also mediate DR-related pathophysiology [104].

### 5.8. Glaucoma

Several possible mechanisms have been explored to determine the underlying pathophysiology of age-dependent glaucoma. For example, failed axonal transport, derived neurotrophic factor, toxic pro-neurotrophins, activated intrinsic and extrinsic apoptotic signals, mitochondrial dysfunction, excitotoxic damage, oxidative stress, pathologically reactive glia, and synaptic failure have been posited [109]. 

Acute severe IOP elevation can cause acute injury and trabecular meshwork fibrosis can cause chronic manifestations [109]. Other possible mechanisms include apoptotic caspase-3, -8, and -9, which are cleaved in RGC after a period of elevated IOP, with inflammatory caspase-1, -4, and -12 also upregulated [60]. Increased AGE accumulations (contributing to increased LC stiffness) and increased RAGE expression on RGC and glial cells were noted in glaucomatous eyes and may propagate neurodegeneration [25]. Optic nerve injury (perhaps due to prolonged elevated IOP) with neuroinflammation and resulting reactive astrocytosis may cause the RGC death observed in glaucoma [110,111]. Glaucoma contributes to retrobulbar optic nerve ischemia with axonal swelling and axonal loss [12,112]; connective tissue in glaucomatous eyes may exhibit ECM modification following stress within or anterior to the LC mediated by integrin-based focal mechanoreceptors [108]. Finally, trabecular meshwork stiffening (evident in glaucoma) along with collagen and ECM accumulation are abundant in presbyopia (age-related vision loss). These suggest possible similarities in pathophysiology between the two diseases [113].

Numerous studies on mitochondrial dysfunction within RGCs and at the ONH have identified structural and biochemical changes that may contribute to glaucoma. Developing pharmacotherapies that equilibrate mitochondrial fusion and fission, address cristae morphological alterations, and eliminate damaged mitochondria via mitophagy may be therapeutically useful [114]. Known molecular targets include DRP1, OPA1, CoQ10, SOD2, and Mic60 (of the mitochondrial contact site and cristae organizing system (MICOS)) [114].

### 5.9. Lack of De Novo RGC Regeneration 

Once RGCs die in the adult stage, they do not regenerate. Recent studies suggest that in vivo reprogramming of Müller glia can convert them into RGCs following downregulation of the expression of Ptbp1, an RNA-binding protein [115]. However, this groundbreaking finding has been called into question due to misinterpretation of results from less-stringently carried out experiments [116,117]. Currently, de novo regeneration of RGCs from in vivo reprogramming when RGCs die after injury or pathological state remains a significant challenge.

## 6. Axon: Age-Dependent Genetic Regulation of Regeneration, Diminished Density, Swelling, and Metabolic Dysfunction

### 6.1. Embryonic

Embryonic RGCs can regenerate axons after injury; embryonic neurons transplanted into an adult CNS, for example, can grow long distances [120]. Neurons lose the ability to intrinsically regenerate post-birth, which may be due to the epigenetic alterations that impact the growth of the optic nerve [121]. Attempts to revitalize adult optic nerve regenerative capacity have not been particularly successful. Ocular diseases affecting the optic nerve, including glaucoma and optic nerve atrophy, are linked to epigenetic dysregulation [88,122,123]. 

Recall normal optic nerve development overproduces axons during the first 15 weeks of gestation. Afterward, approximately 50–70% of axons are eliminated between weeks 16 and 30 of gestation [60,118,119], likely mediated by apoptotic caspases (particularly caspase-3) [60]. 

Mechanistically, deletion of *PTEN*, *IL22*, or *SOCS3* (activate mammalian target of rapamycin (mTOR) and STAT3 pathways) and Notch, Hedgehog (Hh), and mTOR signal pathways (regulate Janus kinase (JAK)/signal transducers and activators of transcription (STAT) pathway) propel cell growth and regeneration; both are minimal after birth in mammals [88,121]. Caspases may also play a role in epigenetic dysregulation. Pharmacological inhibition of caspase-6 and -8, using z-VEID-fmk and z-IETD-fmk, provided RGC neuroprotection and promoted limited RGC axon regeneration, with a few axons extending 0.100 mm beyond the lesion site [60,124]. As caspase-6 is localized to RGC and some microglia, neuroprotective and pro-regenerative impacts of inhibiting caspase-6 are indirectly influenced by ciliary neurotropic factor (*CNTF*) upregulation in retinal glia; the benefits are blocked by suppressing glycoprotein 130 (gp130) and the JAK/STAT pathway [60].

### 6.2. Limitations of Axonal Regeneration

Lack of remyelination is a big barrier to functional RGC axon regeneration. There is no conductivity due to failed remyelination even after the successful regeneration of RGC axons (discussed in the next section “7. Myelin”). 

### 6.3. Loss of Axonal Density

In a widely cited review on human optic nerve aging, Dolman et al. identify age categories most indicative of histologic changes in axonal density [12]. To ensure consistency in the present review, the age cutoffs are reflected here.

#### 6.3.1. Childhood and Adulthood

In humans, investigations report between 770,000 and 1.7 million axonal fibers that are increasingly lost through aging, particularly after age 60 [222]. It has been proposed that there are approximately 170 axons per unit area [12]. 

#### 6.3.2. After Age 60 

The diminished density of axons follows not a linear decline, but a general trend, with loss of axons with increasing age. There is no specific loss of any axonal fiber type [12]. In the same study, individuals did not complain of visual complaints and globes were generally free of disease [12]. In mice, increased ages led to a slightly decreased packing density of axons, increased axon diameter with substantially increased variance, and increased numbers of large and degenerating axons [125]. 

### 6.4. Axonal Swelling

#### 6.4.1. Below Age 65

Histologic studies that reveal swollen axons do not present at the ONH at the level of the cribriform plate, with the exception of patients with polyarteritis [12]; however, more recent studies do report swollen axons in this age group [2].

#### 6.4.2. After Age 70 

Histologic studies of human optic nerves reveal pink, irregular swollen axons at the head of the cribriform plate that stain with hematoxylin and eosin (H&E). Axonal swelling may be due to ischemia, which is likely in elderly individuals due to inadequate posterior ciliary artery perfusion [12]. Glaucoma contributes to retrobulbar optic nerve ischemia with axonal swelling and axonal loss [12,112]. Aged mouse axonal mitochondria experience increased diameter and markedly decreased cristae (in contrast to astrocytic mitochondria, which remain normal throughout life) [125].

Axonal swelling is notable in the human brain with unclear etiology beyond age. These cerebral axonal swellings are believed dystrophic and may be due to the degeneration of cell bodies or transsynaptic degeneration [126]. Swollen axons may be caused by nonvascular, non-glaucomatous senescence and accompanying degradation. For example, in mice, the ultrastructural impacts of age and elevated IOP are comparable, where one month of elevated IOP has similarly destructive effects on the ONH as 18 months of normal aging [125].

The biochemical mechanism of axonal swelling may be due to the activation of nitric oxide synthase (NOS), which is known to cause oxidative and mitochondrial injury in gray matter during ischemia [127]. To identify neuroprotective elements, NOS inhibitors were explored. Notably, Pan-NOS inhibition protected only young axons post-ischemic injury, inhibiting NOS3 protected both young and aging axons, and inhibiting NOS1 selectively protected only aging axons [127]. As aging axons have age-related oxidative injury primarily in white matter, inhibiting glial NOS activity can preserve white matter structure and function [127]. AGEs may also play a role as directly cytotoxic or receptor-mediated signaling (via RAGE) [25,102]. 

### 6.5. Metabolic Dysfunction

Axons experience age-dependent hypometabolism, which leads to significant decreases in maximal respiration, ATP production, and spare capacity [3,130,131]. Studies on the metabolic dysfunction of optic nerve axons have primarily focused on glaucoma, where glaucomatous axonal mitochondria produce more ATP with lower maximal respiration. This may compensate for the axon’s inability to upregulate glycolysis due to lower maximal respiration; however, it is not sufficient for continued function [132]. Altered redox homeostasis is also associated with aging [129], where lowered levels of transcription factor NRF2 limit ROS reduction and result in unchecked oxidative stress [128]. 

#### NAD^+^ and Senescence 

Identified via metabolomics, the “hub” metabolites nicotinamide adenine dinucleotide (NAD^+^), reduced nicotinamide dinucleotide phosphate (NADPH), α-ketoglutarate (αKG), and β-hydroxybutyrate (βHB) are identified aging biomarkers and potential targets for anti-aging pharmacotherapies [129,221]. Notably, NAD^+^ levels decrease with age and have been implicated in neurodegeneration and neuronal death [223]; this may be due to age-dependent declines in neuronal glucose metabolism leading to ATP deficits, subsequently worsened glucose access, limited mitochondrial salvage of NAD^+^, and ultimately Krebs cycle deficiencies [223]. Additionally, NAD^+^ may be a central metabolic marker of senescence, where low NAD/NADH ratios and mitochondrial dysfunction induce senescence [93,221].

## 7. Myelin: Loss of Thickness and Age-Dependent Failure of Remyelination

The present review reflects the age categorization described in Dolman et al., which identified ages of notable histologic change in the human optic nerve.

### 7.1. Newborn

Newborn optic nerves are nearly unmyelinated [12] and experience rapid postnatal visual development, with myelination advancing directionally from the brain to the eye [12,133]. Myelination is primarily mediated by oligodendrocytes [134] with additional involvement from microglia [135,136]. Many epigenomic regulators allow myelination to proceed, including histone deacetylation and repressive histone methyltransferase action [137].

The impact of DNA methylation on myelination cannot be overstated. Myelination is mediated by DNA methylators DNA (cytosine-5)-methyltransferase 1 (DNMT1, *DNMT1*) and DNA (cytosine-5)-methyltransferase 3A (DNMT3A, *DNMT3A*); deletion of these genes in mice resulted in differentiation defects as well as decreased remyelinating capability after injury [137,138,139,140]. Normal myelination in neonates is regulated by DNMT1 and follows the epigenomic regulators of oligodendrocyte precursor cells (OPCs) [88,137]. Ablating the *Dnmt1* gene prevented OPC differentiation via RNA splicing defects (specifically exon-skipping and intron-retention) and endoplasmic reticulum stress; in mice, this triggered ataxia and tremors [137]. In mammals, *DMNT1* deletion is lethal, causing p53-dependent apoptosis and epigenetic deregulation [138,139,140]. Notably, DNMT1 regulation is prominent only in neonate OPCs, with minimal impact on repairing myelin in adult OPCs [88]. Ablating another DNA methyltransferase gene, *Dnmt3A*, in transgenic mice also resulted in oligodendrocyte developmental defects and decreased myelin repair following injury [137]. 

### 7.2. Childhood and Adult

While there is moderate myelination at age two months, human optic nerves do not feature heavy myelin sheaths until age 2, culminating in the dense, thick myelin seen histologically in children and adults ages 4–70 [12]. 

As DNA methylation (mediated by DNMT) is imperative to normal myelination [137], DNA demethylation (mediated by ten–eleven translocation (TET) enzymes) is vital to remyelination [88,141]. Remyelination is mediated by hydroxymethylation and catalyzed by DNA demethylases TET methylcytosine dioxygenase 1 (TET1, *TET1*) and TET methylcytosine dioxygenase 2 (TET2, *TET2*), which are necessary for successful OPC differentiation [88]. (Ablating *Tet2* and *Tet3* in mice also had developmental defects of early-born retinal cell types, including RGCs and amacrine cells [141]). There are higher levels of DNA methylation and hydroxymethylation in adult oligodendrocytes (OL) [88]. 

### 7.3. After Age 70

In aged optic nerves in rats, there is decreased myelin-packing density, morphologically altered myelin, ballooning myelin sheaths, separated myelin lamellae, and oligodendrocyte degeneration [134,142].

OPCs retain their ability to remyelinate during injury, with no epigenomic regulators; however, this intrinsic property worsens with aging [88]. Implicated reasons for this decreased efficiency with aging include ECM remodeling and declining growth factor levels [88]. 

Transcription factor ectopic expression can re-establish epigenetic scenarios and reverse senescence, mediated through Oct4 (encoded by *Pou5f1*), Sox2, and Klf4 (*OSK*) [143]. As these transcription factors support re-establishing epigenetic regulators of remyelination, they provide a hopeful area for basic science and translational research in mitigating the deleterious effects of aging on the optic nerve. 

The senescence-dependent mitigation of hydroxymethylation causes failure of remyelination, which is a significant barrier to functional RGC regeneration [88]. Recent studies have made progress to understand and promote remyelination using the optic nerve crush model in mice [88,144,145]. However, failed remyelination continues to hinder RGC axon regeneration and even leads to the degeneration of RGC axons in nonhuman primates [88,146]. 

## 8. Glial Cell: Reactivity, Remodeling, Hypertrophy, Inclusions, Migration, and Polarization

Optic nerve glial cells are mostly astrocytes, followed by microglia, which may differ in other parts of the CNS [224]. Glial cells (or neuroglia) offer variegated support to neurons, including developmental, physical, immune, repair, homeostatic, metabolic, myelinating, and vascular [136,149]. Intriguingly, aged astrocytes in mice have normal mitochondria, as opposed to pathologic axonal mitochondria that form with aging [125]. Astrocytes can play different roles when they become reactive after injury or in a diseased state, with the capacity to be both beneficial and damaging [149]. Recent investigations into microglia reveal similar dual neuroprotective and neurotoxic capabilities [86]. 

### 8.1. Reactive Astrocytosis

High ROS levels and reduced ATP production lead to a highly inflammatory environment that favors deregulated axonal cell death [150]. There are two types of reactive astrocytes based on etiology: A1 (neural ischemia) and A2 (neural inflammation) [110,149,151]. A1 (ischemic) upregulates neurotoxins along with classical complement genes that interfere with neural synapses; conversely, A2 (inflammatory) upregulates neurotrophic factors [149,151]. These toxic factors released by reactive astrocytes stress RGCs, activate glial cells, and trigger a molecular cascade leading to RGC damage and death [149]. The biochemical mechanisms involve proteins including TGF-β1, TNF, CASP3, and p53 [88,130,147,148,149], which may also underlie the pathogenesis of glaucoma and age-related loss of RGCs [152,153]. Most of the studies currently are limited to rodents; studies using RGCs and astrocytes of human origin are lacking. 

Recently, astrocyte-mediated toxicity in mouse and rat optic nerves was found only following nerve damage [111] in an apparent “two-hit” model of injury and neuroinflammatory mediators. Reactive astrogliosis (without RGC loss) occurred following transiently elevated IOP, systemic inflammatory lipopolysaccharide (LPS) injection, and toxic astrocyte-conditioned media (ACM) [110,111,154,155]; without nerve injury, reactive glial cells and their inflammatory markers do not induce RGC death. Conversely, toxic ACM injection and axotomy by optic nerve crush injury induced RGC death even in reactive-astrocyte deficient *Il1a^−/−^ Tnf^−/−^ C1qa^−/−^* triple knockout (tKO) mice [110,111]. Furthermore, by interfering with glial cell hyperreactivity following damage (using tKO mice or through neutralizing microglial-secreted neuroinflammatory factors IL-1α, TNF-α, and C1q before inducing injury), RGC neurons remained electrophysiologically functional and viable even after optic nerve crush [110,111]. Though studies investigating human reactive astrogliosis are needed, these findings may indicate opportunities for anti-neuroinflammatory therapies to prevent RGC death even after ocular pathology-induced optic nerve injury.

### 8.2. Remodeling

In early axonopathy, astrocyte processes are oriented in parallel, likely due to gap-junction coupling of protein connexin 43 (Cx43) [130]. This parallel formation is also associated with a higher level of intact anterograde transport from the retina to the superior colliculus [130]. 

In later axonopathy, there is the deterioration of organization. Glial coverage increases as axons are lost [130,156]. 

### 8.3. Hypertrophy

Glial cells hypertrophy to fill space from degenerated axons [157]. One mechanism for doing so is through developing abundant glial filaments in processes; specifically, microglial cells become engorged with phagocytosed debris (including degenerated myelin) [72]. Optic nerve head astrocytes (ONHAs) significantly increase oxidative stress on retinal ganglion cell degeneration [157,158]. Estrogen may be protective against neurodegeneration by post-translational modifying tau protein and preventing caspase-3 activation, tau dephosphorylation at Ser422, and tau protein aggregates [158].

### 8.4. Inclusions

#### 8.4.1. Corpora Amylacea (CA)

CA are hallmarks of neurodegeneration and aging [12,159,160]. There remains controversy over the true etiology of CA: are they axonal swellings, or astrocyte destruction? [161,162,163,164] As they do not stain positive for myelin [164], there is strong support that CA indicate astrocyte (as opposed to neuronal) destruction and degeneration [163]. Consistent with neurodegeneration, tau protein has been associated with CA [165]. 

Notably, the largest review on CA to date has suggested renaming these structures to “wasteosomes” to avoid the misconceptions associated with the term amyloid [162] (amyloid signals not only starch-like elements, but also insoluble fibrillary proteins). As CA sequester waste assembled into a glycan structure and secrete these packages outside cells (that are thereafter phagocytosed by macrophages), there is strong evidence that they resemble waste containers that increase with aging [162]. 

#### 8.4.2. Myelin Debris and Lipids

Myelin debris occasionally surrounds CA [159] and is likely from degenerated myelin of optic nerve axons [166]. Lipid inclusions can also be visualized within optic nerve astrocytes and increase with aging [166]. 

### 8.5. Migration

Advanced neurodegeneration suggests possible astrocyte migration, as mediated by integrins [104,108]. 

### 8.6. Microglial Polarization

While astrocytes largely compose glial scars in response to neuronal damage, microglia primarily serve as the CNS’s resident macrophages [136]. The neurotoxic and neuroprotective capabilities of microglia are mediated by two phenotypes that are activated in response to neuroinflammation: M1 (inflammatory, neurotoxic, classically activated) and M2 (anti-inflammatory, neuroprotective, alternatively activated) [86,167,168,169]. Micro-environmental disturbances determine M1/M2 polarization and may depend on MAPK signaling, including JNK inactivation [169]. Balancing microglial M1/M2 polarization has therapeutic potential, where M1 inhibitors alone (including acetaminophen and cyclooxygenase inhibitors such as NSAIDs) neither prevent nor treat neurodegenerative disease [136]. For example, a diet including *Lycium barbarum* (wolfberry or goji berry) boosted M2 polarization, prevented RGC degeneration, and downregulated autophagy following partial optic nerve transection (PONT) in rats [170].

There are many age-dependent changes in microglia, including altered cytokine production, increased activation marker expression, abnormal morphologies, and dynamic behavior changes [136,171]. In comparison with their young counterparts, resting retinal microglia of aged transgenic mice have smaller dendritic arbors with fewer branches [171]. In the same study, injury-associated signaling in aged retinal microglia resulted in decreased process motility and decreased ramification from the resting state, the opposite response of young retinal microglia (which feature fast acute response, increased process motility, increased cellular migration, and increased ramification). Intriguingly, while senescent alterations in microglia result in age-dependent dysregulation and delayed response initiation, the resulting response (once initiated) may be more sustained [171]. 

## 9. Lipofuscin (LF)

Lipofuscin, known as the “wear-and-tear” intracellular hyperpigmentation of post-mitotic cells associated with normal aging, may actively induce pro-inflammatory phenotypes in microglia and astrocytes [172]. In the optic nerve, LF is not easily seen under light microscopy, though it has been noted as electron-dense material mixed with pale droplets in the cytoplasm of astrocytes [12]. LF particles have been detected in aged human optic nerves from healthy donors, as well as in optic nerves from patients with glaucoma and age-related macular degeneration (AMD) [153]. 

Though commonly confused as they are both age-related, lipofuscin differs from drusen (though the presence of LF can exacerbate drusen) [173]. Both hard and soft drusen, rather, are believed to be a combination of lipofuscin and extracellular deposits (therefore containing differing fluorophores [174]) in Bruch’s membrane beneath the RPE [173,175]. Drusen is associated with AMD [175,176] and has varied effects depending on whether drusen is visible or buried in the optic disc [177]. 

## 10. Other Lesions

Vascular degeneration and cavernous degeneration are not explicitly age-dependent changes; however, they are common in elderly individuals and relevant to any discussion of age-related optic nerve neurodegeneration. 

### 10.1. Vascular Degeneration

Vascular degeneration is caused by the hyalinization of arterioles, intimal fibrosis, and elastosis of small arteries. These result in focal scars with complete loss of axons, astrocytic gliosis, and thickening of the septa [12,82]. Other changes include thickened vascular basement membranes and increased deposition of basement membrane collagen [125]. Vascular endothelial cell stress may be induced by integrins α3β1, α6β1, and α6β4, along with α5β1 and αvβ1 [104]. 

### 10.2. Cavernous Degeneration

Cavernous (also known as Schnabel’s cavernous) degeneration is an uncommon histological finding [12]; however, it is highly common in neurodegeneration [178]. Cavernous degeneration can be defined as focal myelin and axonal loss with preserved septa; this causes a spongiform optic nerve proximally [12]. While it was formerly attributed to vascular disease and glaucoma, cavernous degeneration is now thought to be primarily due to vascular disease [178]. 

## 11. Conclusions and Next Steps

Age-dependent changes in the optic nerve greatly alter the risk for ocular pathology throughout the lifespan of a person. Structural changes include connective tissue thickening and stiffening, which lead to decreased optic canal expansion and associated mechanical changes. These physical changes (along with ECM remodeling and molecular changes mediated through genetic regulation, integrins, caspases, and other biochemical mechanisms) lead to RGC loss and decreased axonal regeneration. Even when axons successfully regenerate, however, age-dependent failures of remyelination and senescence prevent conductivity along the axon. Other axonal changes include diminished density, swelling, and metabolic dysfunction. Comparably, glial cell changes (primarily astrocytes, seconded by microglia) cause hyperreactivity, remodeling, hypertrophy, inclusions, migration, and polarization that can cause a highly inflammatory environment that favors RGC axonal cell death. 

Specifically designed investigations on connective tissue, RGCs, axons, oligodendrocytes, and astrocytes from human origin would be meaningful, as would identifying the relative importance of ECM composition and organization at the ONH. Confirming the existence of scleral stiffening, the age-induced elastic fiber quantity alterations at the ONH and LC, and the potential for anti-neuroinflammatory interventions to prevent RGC death remain important challenges to address. Novel therapeutic targets for glaucoma, diabetic retinopathy, and other blinding diseases should consider the mechanisms of aging and associated pathways of neurodegeneration.

## Figures and Tables

**Table 1:** Summary of age-dependent molecular effects on optic nerve (ON) neurodegeneration.

Tissue (Broad)	Tissue (Subtype, Process, Age, or Disease)	Summary of Molecular Mechanisms	Citation
Connective tissue ECM	Elastin	LOX oxidation Decreased quality Further research needed on elastin quantity and relative importance of ECM composition and organization	[12,13,14,15,16,17,18,19,20,21,22,23]
	Collagen	Elevated LOX expression AGE accumulation RAGE “two-hit” hypothesis ECM remodeling Calcium and lipid accumulation MMPs Mechanical considerations Thickening, stiffening	[23,24,25,26,27]
	Dura mater	2nd most ON elastic fibers Calcifications Mechanical considerations Less stiff	[12,16,17,28,29]
	Arachnoid Subarachnoid space	CSFP decreases (linked to normal tension glaucoma) Thickening, more coarse trabeculae, more prominent granulations	[12,30,31,32,33]
	Pia mater Septa	Thickening, expands into fibrous sleeve	[12]
	Lamina Cribrosa (LC)	Most ON elastic fibers Collagen deposition AGE and RAGE ECM remodeling Myofibroblast contraction, TGF-β, mechanotransduction TGF-β1 upregulation MMP-2 Increased cribriform plate rigidity (plastic flow) Unique vascularity (thin and thick beams) Thicker astrocyte basement membranes Integrins α2β1, α3β1, α6β1, and α6β4	[12,18,19,34,35,36,37]
Optic (scleral) canal	Sclera	Decreased elastin Increased collagen AGE Mechanical considerations V-shaped PPS CSCR Further research is needed on scleral stiffening and/or thickening	[31,38,39,40,41,42,43,44,45,46,47,48,49,50,51,52,53,54,55,56,57,58,59]
	Optic nerve head (ONH)	Age-dependent structural changes may alter the biochemical environment RGC loss Smaller optic canal area associated with more axons	[12,20,35,41,60,61,62,63,64,65,66,67]
Retinal ganglion cell (RGC)	Embryonic	Caspase-3 neuronal pruning Histone lysine methylation (H3K9me2, H3K27me3) Histone methyl transferases (G9a (*EHMT2*) and Ezh2 (*EZH2*)) Barhl2 (*BARHL2*) Transcriptional gene control Microglial phagocytosis (complement proteins)	[60,68,69,70,71,72]
	Age-dependent loss	Mechanical and biochemical RGC loss RNFL thinning Caspases (inflammatory, apoptotic) HAT HDAC	[12,60,73,74,75,76,77,78,79,80,81]
	Aging and injury	Upregulated *DLK* CaMKII-CREB signaling interference Caspases HDAC upregulation	[73,75,76,77,78,79,82,83,84,85,86,87,88,89,90]
	Mitochondria	Functional decline ROS Worsened mtDNA repair pathways (lost PARP1, MYH, NTH1) Low NRF2	[91,92,93,94]
	Autophagy	Age-related decline Decreased Atg7 (*ATG7*) and Beclin-1 (*BECN1*) Normal tension glaucoma (*Optn*, *Tbk1*, and *Opa-1*) Autophagy activators (AMPK inducer, mTORC1 inhibitors) may decrease ocular disease severity	[74,95,96,97,98,99,100]
	ipRGC	Decreased density and plexus Lipofuscin accumulation	[74,97,98,99,100]
	Diabetic retinopathy (DR)	Decreased oxygen Abnormal metabolism Polyol and hexosamine pathways DAG-PKC synthesis Free radicals AGE and RAGE Caspases Bax Fas Integrins	[4,5,6,60,61,101,102,103,104,105,106,107]
	Glaucoma	Failed axonal transport Derived neurotrophic factor Pro-neurotrophins Apoptotic signals (intrinsic, extrinsic) Caspases AGE and RAGE Integrins Mitochondrial dysfunction (DRP1, OPA1, CoQ10, SOD2, and Mic60) ECM remodeling Trabecular meshwork stiffening	[12,25,60,108,109,110,111,112,113,114]
	Regeneration	Adult RGCs do not regenerate	[115,116,117]
Axon	Embryonic	Embryonic RGCs regenerate Deletion of *PTEN*, *IL22*, or *SOCS3* (activate mTOR and STAT3 pathways) and Notch, Hh, and mTOR signal pathways (regulate JAK/STAT pathway) propel cell growth and regeneration Caspases	[60,88,118,119,120,121,122,123,124]
	Loss of density	~50–70% of axons eliminated in gestation General (nonlinear) density loss after age 60 May not be associated with visual loss or pathology	[12]
	Swelling	Most notable after age 70 NOS AGE and RAGE	[2,12,25,102,112,125,126,127]
	Metabolic dysfunction	Hypometabolism Decreased respiration, ATP production, spare capacity Altered redox homeostasis Low NRF2 ROS Decreased NAD+ levels may induce senescence	[3,128,129,130,131,132]
Myelin	Newborn	Newborn ON unmyelinated Oligodendrocytes DNA methylation imperative for myelination (DNMT1, DNMT3A)	[12,88,133,134,135,136,137,138,139,140]
	Childhood and adult	Dense myelin sheaths age 2 Remyelination by DNA demethylation, hydroxymethylation (TET1, TET2)	[12,88,137,141]
	After age 70	Decreased myelin packing density Morphological alterations Ballooning sheaths Separated lamellae Oligodendrocyte degeneration ECM remodeling Decreased growth factors Mitigation of hydroxymethylation Oct4 (*Pou5f1*), Sox2, and Klf4 (*OSK*) may contribute	[88,134,142,143,144,145,146]
Glial cell	Reactive astrocytosis	ROS Decreased ATP production Neurotoxins Classical complement Neurotrophic factors TGF-β1, TNF, CASP3, and p53 “Two-hit” model of injury and neuroinflammatory mediators	[88,110,111,130,147,148,149,150,151,152,153,154,155]
	Remodeling	Gap-junction coupling of Cx43 Organizational deterioration Glial coverage increases	[130,156]
	Hypertrophy	Glial filaments in processes Oxidative stress (ROS) Caspases Tau protein Tau dephosphorylation at Ser422	[72,157,158]
	Inclusions	Corpora amylacea (CA) Myelin debris Lipids	[12,159,160,161,162,163,164,165,166]
	Migration	Integrins	[104,108]
	Microglial polarization	M1/M2 polarization imbalance MAPK signaling JNK inactivation Altered cytokine production Increased activation marker expression Abnormal morphologies Dynamic behavior changes	[86,136,167,168,169,170,171]
Lipofuscin (LF)		May actively induce pro-inflammatory glial cell phenotypes	[12,153,172,173,174,175,176,177]
Vascular degeneration *		Hyalinization of arterioles Integrins α3β1, α6β1, and α6β4, along with α5β1 and αvβ1	[12,82,104,125]
Cavernous degeneration *		Common in neurodegeneration Likely due to vascular disease (not glaucoma)	[12,178]

* Not explicitly age-dependent changes, but common in elderly individuals.

## Data Availability

No new data were created or analyzed in this study. Data sharing is not applicable to this article.
